# Renoprotective effects of the methanolic extract of *Tanacetum parthenium* against carbon tetrachloride-induced renal injury in rats

**Published:** 2018

**Authors:** Mohammad Mazani, Yavar Mahmoodzadeh, Mir Mehdi Chinifroush Asl, Shokofeh Banaei, Lotfollah Rezagholizadeh, Alireza Mohammadnia

**Affiliations:** 1 *Department of Biochemistry, School of Medicine, Ardabil University of Medical Sciences, Ardabil, Iran*; 2 *Department of Pathology, School of Medicine, Ardabil University of Medical Sciences, Ardabil, Iran*; 3 *Department of Physiology, Ardabil University of Medical Sciences, Ardabil, Iran*; 4 *Department of Health information technology management, School of medicine, Ardabil University of Medical Sciences, Ardabil, Iran*

**Keywords:** Tanacetum parthenium, Carbon tetrachloride, Antioxidant, Oxidative stress, Kidney

## Abstract

**Objective:**

Studies have demonstrated that carbon tetrachloride (CCl_4_) increases the generation of reactive oxygen species (ROS) in many tissues including the kidney, heart, lung, brain, and liver. The major aim of the present study was to evaluate the protective activity of *Tanacetum parthenium* extract (TPE) in renal tissues of CCl_4_-intoxicated rats.

**Materials and Methods:**

Animals were divided into seven groups of six rats. Group 1 was the control group that was not treated with CCl_4_. The rats in the other groups were intraperitoneally injected with CCl_4_ (1.5 ml/kg, 1:1 in olive oil) on day 14. Rats in the groups bTPE40, bTPE80, and bTPE120 were gavaged with 40, 80, and 120 mg/kg of TPE, respectively for 14 constitutive days on a daily basis, before CCl_4_ administration. Rats in groups aTPE80 and aTPE120 were gavaged with 80 and 120 mg/kg of TPE, respectively, 2, 6, 24 and 48 hr after receiving CCl_4_. Blood samples were collected at the end of the 16^th^ day through an intracardiac puncture and then serums were separated.

**Results:**

CCl_4_ increased urea, creatinine, uric acid and creatinine: albumin (C/A) ratio level in serum and decreased total antioxidant and antioxidant enzymes (SOD and GPx) when compared to the control group (p<0.001). But administration of TPE to rats either before or after exposure to CCl_4_, attenuated these changes when compared with CCl_4_ control group (p<0.05 – p<0.001).

**Conclusion:**

TPE had potent nephroprotective effects against oxygen free radicals produced through CCl_4_ metabolism.

## Introduction

Worldwide, the majority of the people use traditional medicines to cure their illnesses. Plants are regarded as valuable sources for development of new medications by many researchers. Emergence of tolerance towards the existing drugs has attenuated the efficacy of these drugs. Currently, herbal products are widely used to control or prevent the diseases and several plants are being constantly screened in terms of pharmacological activities including antibiotic, anti-apoptotic, antioxidant, and anti-inflammatory properties (Mallhi et al., 2014 ▶). 

*Tanacetum parthenium* L. is a member of the daisy family (Asteraceae); it has more than ten synonyms commonly used in the literature including *Chrysanthemum parthenium* and *Pyrethrum parthenium*. This herb is native to Eurasia and is widely cultivated around the world. Feverfew is a medicinal plant which has been used to reduce fever, asthma, inflammatory conditions, etc. Moreover, it has been employed for the treatment of migraine (Sharopov et al., 2015 ▶). 

Although the phytochemistry of *T. parthenium* has not been studied in detail, major active compounds of this plant are sesquiterpene lactones, parthenolide, 3b-hydroxy parthenolide, canin, and artecanin, having an -methylene butyrolactone moiety. Parthenolide is considered the main biologically active compound of feverfew which is mainly found in the leaves and flower heads (0.20–0.50%) (Jain and Kulkarni, 1999 ▶; Rateb et al., 2008 ▶). 

Many chemicals such as CCl_4_, acetaminophen, and polycyclic aromatic hydrocarbons cause tissue injury in humans and animals. CCl_4_ is well-known for induction of the production of oxygen free radicals in many tissues such as the liver, kidneys, heart, brain, and blood. CCl_4_ is decomposed into trichloromethyl (CCl_3_^.^) and trichloromethyl peroxyl (Cl_3_COO^.^) radicals by the cytochrome oxidase enzyme complex. These free radicals cause cytoplasmic membrane phospholipids to undergo lipid peroxidation. Functional changes occur in the cell membrane as a result of lipid peroxidation (Aksoy and Sözbilir, 2012 ▶). 

Antioxidants prevent this chain reaction of lipid peroxidation. Moreover, by scavenging oxygen free radicals, antioxidants play an important role in protecting the kidneys against oxygen free radicals (Zirak et al., 2014 ▶). Living organisms possess endogenous antioxidant defense systems. Superoxide dismutase (SOD), glutathione (GSH), glutathione peroxidase (GPx), glutathione-S-transferase (GST), and catalase (CAT) are endogenous antioxidants. It has been reported that many herbal products have protective effects against renal injury and have antioxidant activities (Aksoy and Sözbilir, 2012 ▶). Although favorable ethnopharmacological properties of *T. parthenium* extract (TPE) have been shown, its protective effect against CCl_4_-induced nephrotoxicity has not been previously explored. In this study, CCl_4_-induced renal injury was induced in rats and the impact of increasing doses of *T. parthenium* against the damage was investigated. For this purpose, the levels of urea, creatinine, albumin, creatinine: albumin ratio and uric acid were evaluated in the serum, malondialdehyde (MDA), SOD, GPx and total antioxidant levels in renal tissue were also measured.

## Materials and Methods

**Chemicals**

Commercial kits for determination of SOD and GPx levels were purchased from Randox Laboratories Ltd (Crumlin, UK) and commercial kits for determination of urea, creatinine, and albumin levels were purchased from Parsazmun Laboratory (Tehran, Iran). Thiobarbituric acid, trichloroacetic acid, bovine serum albumin, sodium acetate, 3H_2_O, CCl_4_, Ferric chloride (FeCl_3_) and Ferrous sulphate (FeSO_4_) were purchased from Merck (Germany) and 2,4,6-tri[2-pyridyl]-s-triasine (TPTZ) was purchased from Fluka (Sigma, Germany). All other chemicals and reagents used in this study were of analytical grade. 

**Collection and authentication of plant**

Aerial parts of *T. parthenium* were collected from the Vigan village, Arasbaran region, Iran, 2100 m above sea level, during its flowering season on May 5, 2015. The plant was authenticated by experts in the herbarium of Eastern Azerbayjan natural resource and agriculture research center with herbarium No. 2411; then, it was prepared for extraction.

**Preparation of plant extract**

The aerial parts of *T. parthenium* were washed with tap water and then air-dried. The dried plant was pulverized into a coarse powder by a mechanical grinder and stored in an air-tight container. Dried powder (1 kg) was soaked in an aqueous methanol solution (30:70) for ten days with daily shaking. After this period, the extract was filtered and concentrated using a rotary evaporator at 45^º^ C. Then, it was converted to a dry powder using freeze-dryer for two weeks in 3 steps: freezing, main drying, final drying. The crude extract was stored in an air-tight container. The methanolic extract of *T. parthenium* was prepared fresh each time using distilled water immediately before the administration.

**Experimental animals**

In this study, 42 male wistar rats, weighing between 180±20 g, were used. After randomization into various groups, the rats were acclimatized for a period of 7 days under standard conditions at room temperature (25±3^º^ C) with 12 hr/ 12 hr light/dark cycles. All the animals were fed under strict hygienic conditions with rodent pellet diet and water *ad libitum*. The animal experiments ethics committee approved the study (Approval number: IR.ARUMS.REC.1394.67). The study was conducted in accordance with the Guide for the Care and Use of Laboratory Animals published by the US National Institutes of Health. 

**Experimental design**

Group I served as normal control for both prophylactic and curative studies and received oral distilled water for 14 days and on the 14th day, they were treated with olive oil (1.5 ml/kg, i.p.). Group II served as a toxic control with CCl_4_ for both prophylactic and curative studies and received oral distilled water for 14 days and on the 14th day, they were treated with CCl_4_ (1.5 ml/kg i.p.) diluted (1:1) with olive oil. 

**Figure 1:**
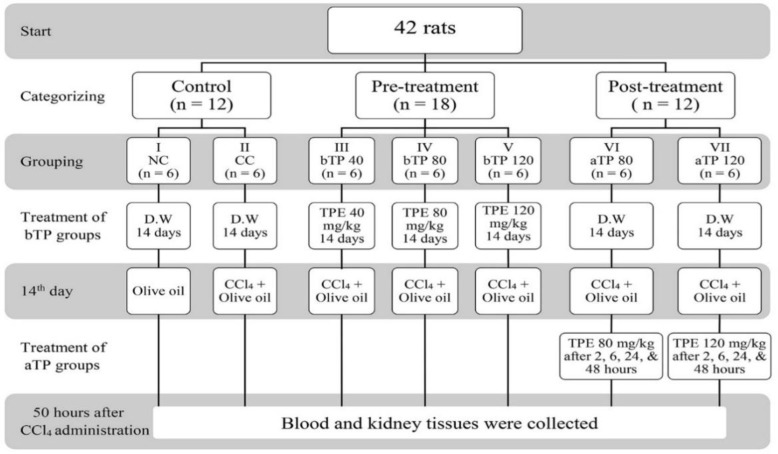
Flow diagram of the experimental design. NC: Normal control, CC: CCl_4_ control, bTP40: Pre-treatment with 40 mg/kg TPE, bTP80: Pre-treatment with 80 mg/kg TPE, bTP120: Pre-treatment with 120 mg/kg TPE, aTP80: Post-treatment with 80 mg/kg TPE, and aTP120: Post-treatment with 120 mg/kg TPE. D.W: Distilled water

Group III, IV, and V served as pre-treatment groups (prophylactic). They received oral methanolic extract of *T. parthenium* 40, 80, and 120 mg/kg for 14 days, respectively, and on the 14^th^ day, animals received CCl_4_ (1.5 ml/kg i.p.) diluted (1:1) with olive oil, 2 hr after administration of the last dose of the extract. Group VI and VII served as post-treatment groups (curative). They received oral distilled water for 14 days and on the 14th day, they received CCl_4_ (1.5 ml/kg i.p.) diluted (1:1) with olive oil, followed by oral administration of methanolic extract of *T. parthenium* at the doses of 80 mg/kg (Group VI) or 120 mg/kg (Group VII) 2, 6, 24, and 48 hr after CCl_4_ intoxication. All rats were sacrificed 50 hr after CCl_4_ administration. Just before sacrifice, blood was collected from the heart under mild ketamine anesthesia (Figure 1).

**Biochemical investigation**

*Renal function tests*

 Here, 50 hours after the CCl_4_ injection, all the animals were anesthetized using ketamine 10% and sacrificed. The blood was collected and serum was separated by centrifugation (Eppendorf, 5810 R) at 3000 rpm for 10 min. Serum albumin, urea, creatinine, and uric acid levels were assessed calorimetrically using commercial diagnostic kits obtained from Parsazmun, Iran. Then, creatinine/albumin ratio (C/A) was calculated (Zeinali et al., 2017 ▶). All serum parameters were measured using a spectrophotometer (Eppendorf, Ecom-E6125). 

*Kidney homogenates*

 After killing the rats, their kidney tissues were dissected out, washed with ice-cold normal saline to completely remove all the blood cells. Then, samples were cut into small pieces, one piece placed in 50 mM Tris buffer (pH 7.4) and homogenized using Heidolph (Silent Crusher) homogenizer to obtain 10% homogenates. The homogenate was centrifuged at 3000 rpm for 15 min. The supernatant was collected,transferred to an Eppendorf tube and centrifuged at 12000 rpm for 20 min (Eppendorf, 5810 R). The supernatant was used for the determination of MDA (as a lipid peroxidation marker), total antioxidant, superoxide dismutase (SOD) and glutathione peroxidase (GPx) levels. 

**Determination of MDA level**

Lipid peroxidation in the kidney homogenate was expressed in terms of thiobarbituric acid reactive substances (TBARS) by measuring malondialdehyde (MDA) level, spectrophotometrically (Eppendorf, Ecom-E6125) in kidney homogenates according to Mihara and Uchiyama (Uchiyama and Mihara, 1978). Briefly, 0.5 ml of the supernatant of kidney homogenates was mixed with 2400 l of 1% aqueous orthophosphoric acid solution and 1 ml of 0.67% thiobarbituric acid (TBA) solution in water, and heated in a boiling water bath for 45 min. The pink-colored chromogen formed by the reaction of TBA with MDA, was extracted by n-butanol and measured at 532 nm. MDA levels were expressed as nmol/mg protein.

**SOD and GPx measurement**

Commercial kits were used to determine SOD and GPx activities (Randox Laboratories, UK). In the SOD determination method, uric acid and superoxide radicals are produced from xanthine with the reaction being catalysed by xanthine oxidase at 505 nm. The superoxide radicals produced in this reaction react with 2-(4-iodophenyl)-3-(4-nitrophenyl) -5- phenyl tetrazolium chloride (INT) to produce red formazon. Tissue SOD enzyme activity correlates with the degree of inhibition of this reaction. In the GPx determination method, oxidized glutathione is reduced by glutathione reductase in the presence of NADPH. At the same time, NAPDH is oxidized to NADP‏. The change in absorbance at 340 nm is dependent on the decrease in reduced NADPH measured spectrophotometrically (T80+UV/Vis spectrometer PG Instrument).

**Assay of total antioxidant capacity (TAC**)

Total antioxidant capacity was measured by ferric reducing ability of tissue (FRAP) method. This method is based on the ability of tissue in reducing Fe ^3+^ to Fe ^2+^ in the presence of TPTZ. The reaction between Fe ^2+^ and TPTZ gives a blue complex with the maximum absorbance at 593 nm (Benzie and Strain, 1996 ▶). 

**Total protein measurement**

Protein concentrations in the samples were measured by the Bradford method using concentrated Coomassie blue reagent. Bovine serum albumin was used as the standard (Bradford, 1976 ▶).

**Histopathological studies**

The kidney tissue was collected and fixed in 10% formalin, dehydrated in ethanol (50–100%), cleared in xylene, and embedded in paraffin. Sections of 4–5 μm thickness were prepared, stained with hematoxylin and eosin (H&E) dye and examined for histopathological changes under the microscope (Olympus IX71).

**Statistical analysis**

Data are presented as mea ± standared diviation (SD). Statistical analysis was performed using one-way analysis of variance (ANOVA) followed by *post hoc *LSD test to correct for multiple comparison treatments, using the SPSS for Windows statistical package, version 16.0. A p<0.05 was considered significant. Graphs were plotted using Microsoft Excel 2013. 

## Results

**Effect of** ***T. parthenium*** **extract on serum biochemical parameters**

The renoprotective effects of *T. parthenium* extract (TPE) on serum biochemical parameters in CCl_4_-intoxicated rats are shown in Table. 1. Rats treated with CCl_4_ (Group II) showed a significant increase in serum urea, creatinine, uric acid and creatinine: albumin ratio and a significant reduction in albumin levels was observed compared to control animals (Group I) (p<0.001). Pre-treatment with *T. parthenium* extract at 40, 80 and 120 mg/kg for 14 days (Groups III, IV and V, respectively) and post-treatment with *T. parthenium* extract at 80 and 120 mg/kg (Groups VI and VII, respectively) , 2, 6, 24 and 48 hr after CCl_4_ intoxication showed renal protection in terms of serum urea, creatinine and creatinine: albumin proportion levels compared to the toxic control group (Group II) (p<0.001).

**Table 1:** Effect of *Tanacetum parthenium* extract on biochemical indices in rats treated with CCl_4_

**Groups**	**Urea** (mg/dl)	**Creatinine** (mg/dl)	**Uric acid** (mg/dl)	**albumin** (mg/dl)	**Creatinine/ albumin**
**NC**	44.16±6.08	0.36±.03	1.16±0.16	2.9±0.11	0.12±.01
**CC**	58.42±7.2•	1.12±0.18 •	2.01±0.23 •	1.8±0.13 •	0.62±0.1 •
**bTP40**	53.33±5.8	0.98±0.07 *	1.78±0.42	1.89±.08	0.52±.02 **
**bTP80**	51.16±7.8*	0.83±0.05**	1.7±0.36	2.02±0.21 *	0.41±.02 **
**bTP120**	49.66±6.4 *	0.76±0.05 **	1.63±0.3 *	2.15±0.23 **	0.36±.05 **
**aTP80**	51.8±3.8	0.93±0.05 **	1.8±0.23	1.94±.08	0.48±.04 **
**aT120**	50.3±4.6*	0.83±0.04 **	1.71±0.19	2.03±0.15 *	0.41±.04 **

Values are presented as mean±SD (n=6).

NC: Normal control, CC: CCl_4_ control, bTP40: Pre-treatment with 40 mg/kg TPE, bTP80: Pre-treatment with 80 mg/kg TPE, bTP120: Pre-treatment with 120 mg/kg TPE, aTP80: Post-treatment with 80 mg/kg TPE, and aTP120: Post-treatment with 120 mg/kg TPE.

• Indicates significant differences (p<0.001) compared to the normal control group (NC).

* indicates significant differences (p<0.05) compared to the CCl_4_ control group (CC).

** indicates significant differences (p<0.001) compared to the CCl_4_ control group (CC).

**Effect of** ***T. parthenium*** **extract on lipid peroxidation**

**Figure 2:**
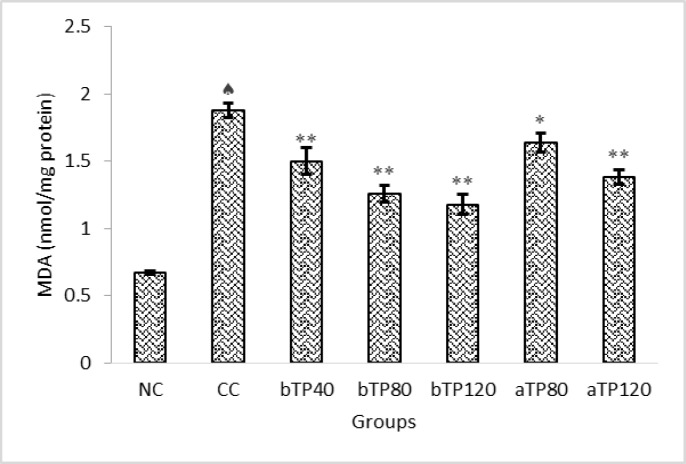
Effect of TPE on kidney lipid peroxidation in CCl_4_-intoxicated rats.   Indicates significant differences (p<0.001) compared to the normal control group (NC).   indicates significant differences (p<0.05) compared to the CCl_4_ control group (CC). ** indicates significant differences (p<0.001) compared to the CCl_4_ control group (CC). NC: Normal control, CC: CCl_4_ control, bTP40: Pre-treatment with 40 mg/kg TPE, bTP80: Pre-treatment with 80 mg/kg TPE, bTP120: Pre-treatment with 120 mg/kg TPE, aTP80: Post-treatment with 80 mg/kg TPE, and aTP120: Post-treatment with 120 mg/kg TPE. MDA: Malondialdehyde. Data are presented as mean ± SD

Lipid peroxidation increased in the CCl_4_ control group, as indicated by elevated MDA levels, when compared to the normal control group. Pre- and post-treatment with TPE decreased the MDA levels which was significantly more pronounced at the higher dose (Figure 2) (p<0.05 – p<0.001).

**Effect of** ***T. parthenium*** **extract on antioxidants levels**

**Figure 3:**
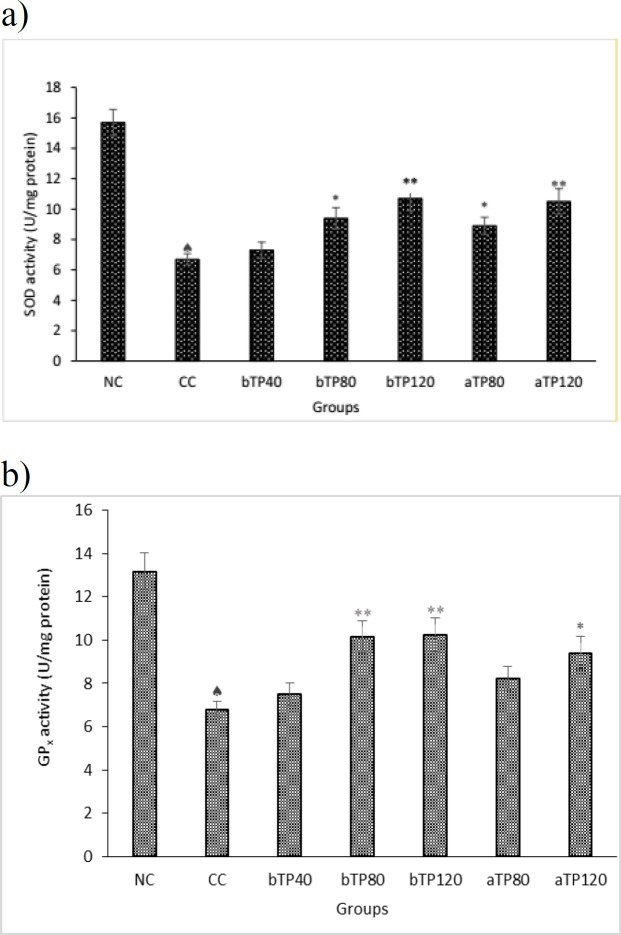
a) Effect of TPE on kidney SOD activity in CCl_4_-intoxicated rats.   Indicates significant differences (p<0.001) compared to the normal control group (NC). * indicates significant differences (p<0.05) compared to the CCl_4_ control group (CC). ** indicates significant differences (p<0.001) compared to the CCl_4_ control group (CC). NC: Normal control, CC: CCl_4_ control, bTP40: Pre-treatment with 40 mg/kg TPE, bTP80: Pre-treatment with 80 mg/kg TPE, bTP120: Pre-treatment with 120 mg/kg TPE, aTP80: Post-treatment with 80 mg/kg TPE, and aTP120: Post-treatment with 120 mg/kg TPE. SOD: Superoxide dismutase

**Figure 4:**
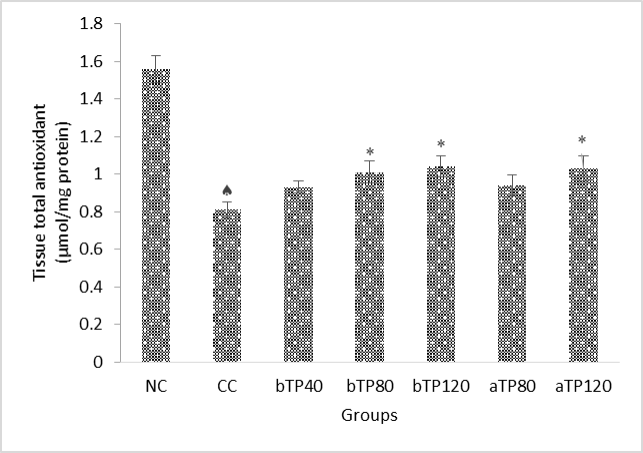
Effect of TPE on kidney total antioxidant in CCl_4_-intoxicated rats.   Indicates significant differences (p<0.001) compared to the normal control group NC). * indicates significant differences (p<0.05) compared to the CCl_4_ control group (CC). NC: Normal control, CC: CCl_4_ control, bTP40: Pre-treatment with 40 mg/kg TPE, bTP80: Pre-treatment with 80 mg/kg TPE, bTP120: Pre-treatment with 120 mg/kg TPE, aTP80: Post-treatment with 80 mg/kg TPE, and aTP120: Post-treatment with 120 mg/kg TPE. Data are presented as mean ± SD

Administration of CCl_4_ drastically decreased the tissue total antioxidant, as well as SOD and GPx levels (p<0.001). Pre-treatment with 40 mg, 80 mg and 120 mg/kg and post-treatment with 80 mg/kg and 120 mg/kg of *T. parthenium* extract caused a significant increase in tissue total antioxidant, SOD and GPx levels and restored them to levels near those of the normal controls, and this increase was greater in rats receiving the higher dose of TPE. (p<0.05 – p<0.001) (Figures 3 and 4).

b) Effect of TPE on kidney GP_x_ activity in CCl_4_ -intoxicated rats.   Indicates significant differences (p<0.001) compared to the normal control group (NC). indicates significant differences (p<0.05) compared to the CCl_4_ control group (CC). ** indicates significant differences (p<0.001) compared to the CCl_4_ control group (CC). NC: Normal control, CC: CCl_4_ control, bTP40: Pre-treatment with 40 mg/kg TPE, bTP80: Pre-treatment with 80 mg/kg TPE, bTP120: Pre-treatment with 120 mg/kg TPE, aTP80: Post-treatment with 80 mg/kg TPE, and aTP120: Post-treatment with 120 mg/kg TPE. GP_x_: Glutathione peroxidase. Data are presented as mean ± SD.

**Effect of** ***T. parthenium*** **extract** **on histopathological change in the kidney**

**Figure 5:**
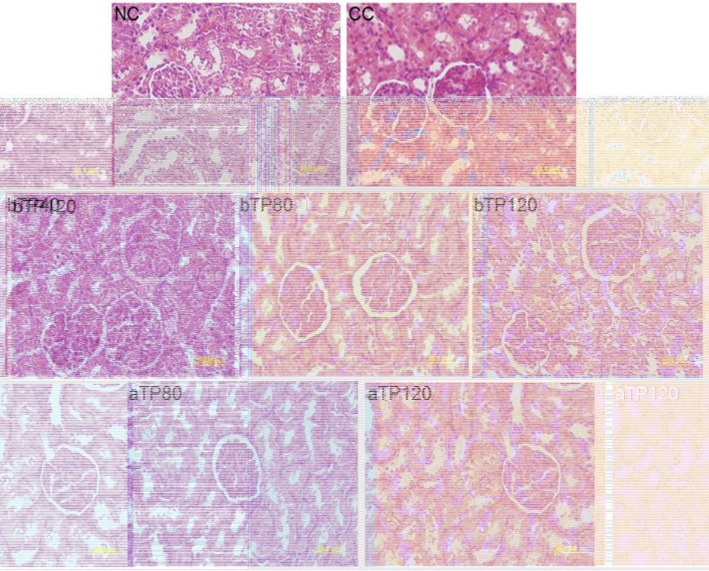
The photomicrographs of kidney sections from rats treated with CCl_4_, pretreated with TPE at 40, 80, and 120 mg/kg, post-treated with 80 and 120 mg/kg and vehicle. Kidney tissues were stained with H&E. NC: Normal control, CC: CCl_4_ control, bTP40: Pre-treatment with 40 mg/kg TPE, bTP80: Pre-treatment with 80 mg/kg TPE, bTP120: Pre-treatment with 120 mg/kg TPE, aTP80: Post-treatment with 80 mg/kg TPE, and aTP120: Post-treatment with 120 mg/kg TPE

Figure 5 illustrates the histopathological changes in the kidney. Kidney damage was evidenced by preliminary signs of acute tubular necrosis (ATN), histological alterations in proximal and distal tubules and inflammation in the tubules. These changes significantly reduced in the TPE-treated group. The changes were completely absent in normal controls. The kidney of the control group showed normal glomerular (NG). However, CCl_4_-treated rat kidneys showed focal hyaline cast and inflammation in the tubules. Following treatment with TPE, the kidney only showed hemorrhage and cloudy swelling (bTP40, bTP 80, bTP120, aTP80 and aTP120 mg/kg). 

## Discussion

Antioxidants are vital substances which can protect the body from injuries caused by free radicals. This antioxidant capacity of chemicals can be beneficial for development of novel medicines against degenerative diseases. Based on the growing interest in free radicals biology and the usefulness of antioxidants in protection against oxidative stress, antioxidants are regarded as a tool to prevent or slow down the progression of conditions attributed to oxidative stress (Khan et al., 2010 ▶).

A substantial number of herbal formulations has been shown to have therapeutic properties against several life-threatening diseases (Hussain et al., 2014 ▶). The plants that exhibit tissue protective effects, have the phytoconstituents such as phenyl compounds, coumarins, essential oils, steroids, alkaloids and other nitrogenous compounds that possess protective effects by preventing the tissue from the damaging effects of toxins (Hussain et al., 2014 ▶; Pareek et al., 2011 ▶; Sharopov et al., 2015 ▶). 

In the present study, administration of CCl_4_ caused nephrotoxicity as indicated by the elevation in serum levels of urea and creatinine. 

The elevation in the plasma creatinine and urea levels can indicate acute kidney injury (Pourfarjam et al., 2017 ▶). In addition, decrease in plasma albumin concentration in CCl_4_-treated rats might have resulted from remarkable leakage due to hypercellularity of both glomeruli and tubules. Treatment with TPE significantly improved the concentration of albumin in plasma; also, a significant recovery was noticed in the levels of urea, creatinine, and creatinine/albumin ratio. This effect may be related to the antioxidant properties of TPE since it has been found that ROS may impair glomerular filtration rate (El-mohsen Ali and Abdelaziz, 2014 ▶). 

Lipid peroxidation is an important marker of oxidative stress (Vuda et al., 2012 ▶). The increase in renal MDA levels induced by CCl_4_ suggests increased lipid peroxidation, leading to the failure of antioxidant defense mechanisms that prevent formation of excessive free radicals (Ganie et al., 2011 ▶). Free radical scavenging is one of the major antioxidation mechanisms inhibiting the chain reaction of lipid peroxidation (Vuda et al., 2012 ▶). In the present study, pre- and post-treatment with the methanolic extract of *T. parthenium* reduced the lipid peroxidation by decreasing MDA levels, indicating the free radical scavenging activity of this plant extract under *in vivo* conditions. We further studied the *in vivo* antioxidant activity of the methanolic extract of *T. parthenium* by estimation of renal total antioxidant, SOD and GPx levels. Previous studies on the mechanism of CCl_4_-induced tissue damage indicated that antioxidant enzymes such as SOD and GPx play a key role in detoxification of toxic metabolites (Mahmoodzadeh, Mazani, and Rezagholizadeh, 2017 ▶). In addition to non-enzymatic antioxidants, enzymatic antioxidants also play important roles in protecting cells against oxidative damage. These enzymes combat free radicals. SOD is an important defense enzyme that catalyses the dismutation of superoxide anions. GPx is an enzyme that causes hydroperoxide to decompose into non-toxic products by H_2_O_2_ reduction (Aksoy and Sözbilir, 2012 ▶). 

CCl_4_-induced oxidative stress in renal tissues led to the increase in hydrogen peroxides and superoxides levels. In our study, it is evidenced by the decline in the activities of SOD and GPx in the kidney tissue (El-mohsen Ali and Abdelaziz, 2014 ▶). These results confirm other findings and are also in accordance with the data reported by Venkatanarayana et al. which showed that the activities of antioxidant enzymes markedly decreased in kidney tissue homogenates of rats treated with CCl_4_ (Sahreen et al., 2015 ▶; Venkatanarayana et al., 2012 ▶). Concomitant administration of TPE with CCl_4_ significantly decreased the toxic effect of CCl_4_ on the activities of these enzymes.

The histopathological results showed marked pathological changes in the kidney of CCl_4_-treated rats. In our study, CCl_4_-treated rats showed the preliminary signs of acute tubular necrosis (ATN), histological alterations in proximal and distal tubules and inflammation in the tubules. Regenerative changes in the glomerulus and convoluted tubules were observed in TPE-treated rats. This histopathological effect may be related to the presence of antioxidant materials in TPE.

Flavonoids are important compounds present in plants and have been previously reported to have renoprotective activities (Zeinali et al., 2017 ▶). The qualitative determination of these flavonoids by HPLC confirmed the presence of kaempferol, flavonol, fisetin, apigenin, and naringenin (Shafaghat and Salimi, 2008 ▶). Kaempferol and apigenin have already been reported to have antioxidant activity (Mallhi et al., 2014 ▶). Therefore, renoprotective activity of the TPE may be due to the presence of these compounds (Mallhi et al., 2014 ▶).

The results obtained in this study suggest that the protective effects of TPE against CCl_4_-induced oxidative stress, could be attributed mainly to the presence of high content of phenolics and flavonoids which have profound antioxidant activity. These compounds could scavenge the free radicals generated from CCl_4_ through cytochrome P450 enzyme system, and thereby diminished the oxidative injuries. Histopathological examinations data are in agreement with biochemical analysis findings.
